# Specific molecular imaging of BALB/c model mice with Graves’ ophthalmopathy based on high expression of insulin-like growth factor 1 receptor

**DOI:** 10.1007/s12149-024-02013-4

**Published:** 2025-02-07

**Authors:** Zhiting Zhang, Ziyu Ma, Xuan Wang, Yaqian Zhou, Ruixin Wu, Yiming Shen, Ning Li, Qiang Jia, Hong Zhang, Wei Li, Wei Zheng

**Affiliations:** 1 Tianjin Medical University General Hospital, Tianjin, China; 2 Tianjin Chest Hospital, Tianjin, China; 3 Renji Hospital, Shanghai Jiao Tong University School of Medicine, Shanghai, China; 4 Shanghai Tenth People’s Hospital, Shanghai, China

**Keywords:** Graves’ disease, Graves’ ophthalmopathy, IGF-1R, Peptide probe, Z_IGF1R:4551_-GGGC, TSHR

## Abstract

**Objective:**

At present, most of the targeted imaging based on insulin-like growth factor 1 receptor (IGF-1R) is for tumor research, and there is no IGF-1R-targeted imaging for Graves’ ophthalmopathy(GO). This study aims to develop a peptide probe, ^99m^Tc-Z_IGF1R:4551_-GGGC, targeting the IGF-1R, and to achieve specific imaging in Graves’ disease (GD) animal models exhibiting GO.

**Methods:**

99mTc-ZIGF1R:4551-GGGC probe was synthesized using a direct labeling method and its labeling efficiency assessed via instant thin-layer chromatography (ITLC). Western blot analysis confirmed the overexpression of IGF-1R in malignant melanoma B16F10 cells. Subsequent SPECT/CT whole-body imaging of B16F10 tumor-bearing mice evaluated the probe’s targeting accuracy. In addition, a GO model was established using an electroporation immunoassay, followed by serological and histopathological examinations. The GO models then underwent 99mTc-ZIGF1R:4551-GGGC SPECT/CT imaging to assess eye-targeted imaging capabilities.

**Results:**

The peptide probe exhibited a labeling efficiency exceeding 90%. Both GD and GO models were effectively created via electroporation immunoassay. Imaging results indicated significant accumulation and retention of the peptide probes in the tumors of B16F10 tumor-bearing mice. In the GO models, probe uptake was predominantly observed in retrobulbar tissues, contrasting with primary accumulation in the lungs and gastrointestinal tract in normal mice, where only minimal tracer was observed in retrobulbar tissues. Notably, GO mice demonstrated higher probe uptake and prolonged retention.

**Conclusion:**

This study successfully established GD and GO models, reducing the duration of the immune cycle. Moreover, a peptide probe targeting IGF-1R was synthesized, enabling specific imaging of retrobulbar tissues in GO models.

## Introduction

The etiology of Graves’ disease (GD) with Graves’ ophthalmopathy (GO) is multifaceted and complex. GO primarily arises from inflammatory infiltration, fat accumulation, and muscle tissue expansion [1]. Recent studies have highlighted the co-localization of thyrotropin receptor (TSHR) and insulin-like growth factor 1 receptor (IGF-1R) in thyroid cells and orbital fibroblasts in GO patients. This co-localization is crucial in mediating the proliferation of orbital fibroblasts, playing a pivotal role in the onset and progression of GO [1–4]. IGF-1R, in particular, is an initiating factor and a significant pathogenetic agent in GO development [3, 4]. Consequently, current research is increasingly focused on unraveling the pathogenesis of GO and developing specific imaging techniques centered on IGF-1R targets, aiming to facilitate early diagnosis, treatment, and disease monitoring in GO patients.

IGF-1R, a member of the tyrosine kinase receptor family, is a bilayer transmembrane receptor implicated in the pathogenesis of various malignancies [5, 6]. Studies have shown a strong correlation between the abnormal overexpression of IGF-1R and both tumor metastasis and resistance to treatment [7]. Keeping this in the background, Tolmachav et al., developed ^99m^Tc-Z_IGF1R:4551_-GGGC peptide probe which was characterized as having a small size, high affinity, and enhanced targeting specificity [6]. Therefore, to document the role of IGF-1R in GO development, the potential of ^99m^Tc-Z_IGF1R:4551_-GGGC a SPECT imaging probe was investigated in the present study.

The foundational approach to constructing an animal model of GO involves immunizing mice with the TSHRα subunit, thereby inducing the production of thyrotropin receptor antibodies (TRAb) [8]. In our previous research, we successfully established a GO model using the recombinant plasmid pcDNA3.1/TSHR289 [9]. In addition, we have effectively extracted the IGF-1Rα subunit recombinant plasmid pcDNA3.1/IGF-1Rα [10]. By leveraging the co-pathogenic mechanism of TSHR and IGF-1R, we co-injected these recombinant plasmids into BALB/c mice, successfully creating a more stable and reliable GO model for specific imaging studies [11].

## Materials and methods

### Peptide probe

#### Synthesis and labeling

The synthesis of Z_IGF1R:4551_-GGGC was commissioned by Qiang Yao Biological Company (Shanghai, China). The synthesis of the radiolabeled product ^99m^Tc-Z_IGFIR-4551_-GGGC was performed and the methodology has been reported previously [6]. The labeling rate was determined by ITLC.

#### Validation of specificity

**Fig. 1:**
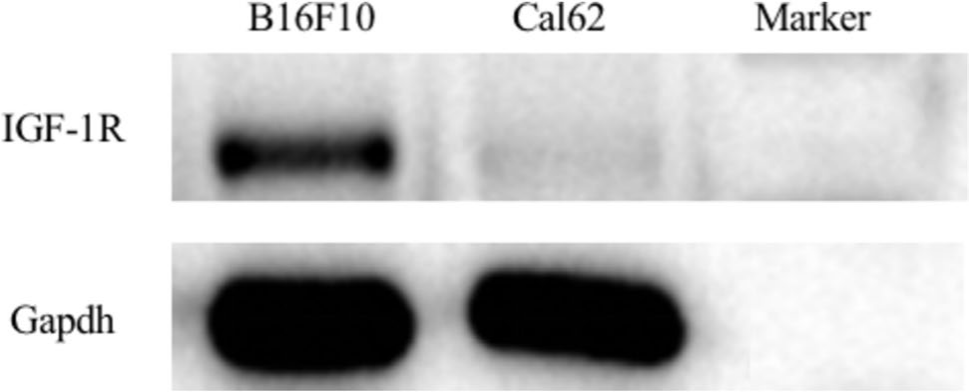
Results of IGF-1R expression in B16F10 and Cal62 cells

Malignant melanoma cells B16F10 and thyroid cancer cells cal62 were cultured. B16F10 is characterized by high expression of IGF-1R, while cal62 is the opposite, so we used these two cells to verify the specificity. Western blotting was used to verify the high expression of IGF-1R in B16F10, as illustrated in Fig. 1. C57BL/6 female mice were used to construct B16F10 tumor-bearing mice, which were randomly divided into an experimental group and a control group, with 6 mice in each group. The experimental mice were injected with 300 μCi ^99m^Tc-Z_IGF1R:4551_-GGGC by intra-tumor injection. Control mice were injected with 300 μCi ^99m^TcO_4_^−^ in the same way. SPECT/CT whole-body imaging was performed at 0.5 h and 4 h after injection, respectively.

### Construction and verification of GO model mice

The experimental procedures on animals was carried out in accordance with the current ethical guidelines and approved by the Animal Welfare and Ethics Committee of Tianjin Medical University (Ethical NO. IRB2022-WZ-153).

**Fig. 2:**
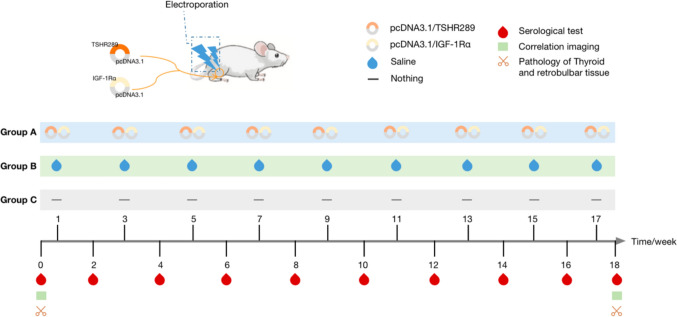
Grouping and related operations of BALB/c mice

The recombinant plasmids pcDNA3.1/TSHR289 and pcDNA3.1/IGF-1Rα were extracted according to the previous research methods of our team [9–11]. The time points involved in the construction and validation of animal models are set based on the results of previous studies [11]. Twenty-four BALB/c female mice (6-week-old) were randomly divided into group A (experimental group), group B (control group) and group C (blank group), with 8 mice in each group, and constructed according to the scheme shown in Fig. 2.

For group A, two recombinant plasmids (50 μl each) were injected into the gastrocnemius muscle of both hind limbs at the same time, and electroporation was performed at the injection site immediately. Group B was injected with saline, and the other operations were the same as group A. No processing is performed in group C. Each group was immunized once every 2 weeks for a total of nine times. Mice were weighed and tested for Thyroxine (T_4_), thyroid-stimulating hormone (TSH) and thyrotropin receptor antibody (TRAb) before the first immunization and 1 week after each immunization (i.e., weeks 0, 2, 4, 6, 8, 10, 12, 14, 16, and 18). Imaging was performed before the first immunization and 1 week after the last immunization (i.e., week 0 and week 18). One week after the end of the last immunization (i.e., week 18), some mice were randomly killed, and pathological analysis of thyroid, eyeball and retrobulbous tissue was performed.

### Specific imaging of GO model mice

^99m^TcO4^−^ thyroid imaging and orbital MRI were performed in groups A, B and C before the first immunization and 1 week after the last immunization (i.e., week 0 and week 18). Upon successful construction of the GO model, ^99m^Tc-Z_IGF1R:4551_-GGGC-specific imaging was performed. The imaging agent was injected into the right inner canthal vein with a 1 ml insulin needle. First, group A was randomly divided into two groups and received 300 μCi (75 μl) of ^99m^Tc-Z_IGF1R: 4551_-GGGC and 300 μCi (75 μl) of ^99m^TcO_4_^−^, respectively. Group B received 300 μCi (75 μl) of ^99m^Tc-Z_IGF1R: 4551_-GGGC. SPECT/CT whole-body imaging was then carried out at 0, 3, and 6 h post-injection.

### Statistical analysis

All data are expressed as mean ± standard deviation. One-way ANOVA or independent sample *t* test was used for comparison between the groups. When *P* < 0.05, the difference was statistically significant.

## Results

### Peptide probe and labeling

**Fig. 3:**
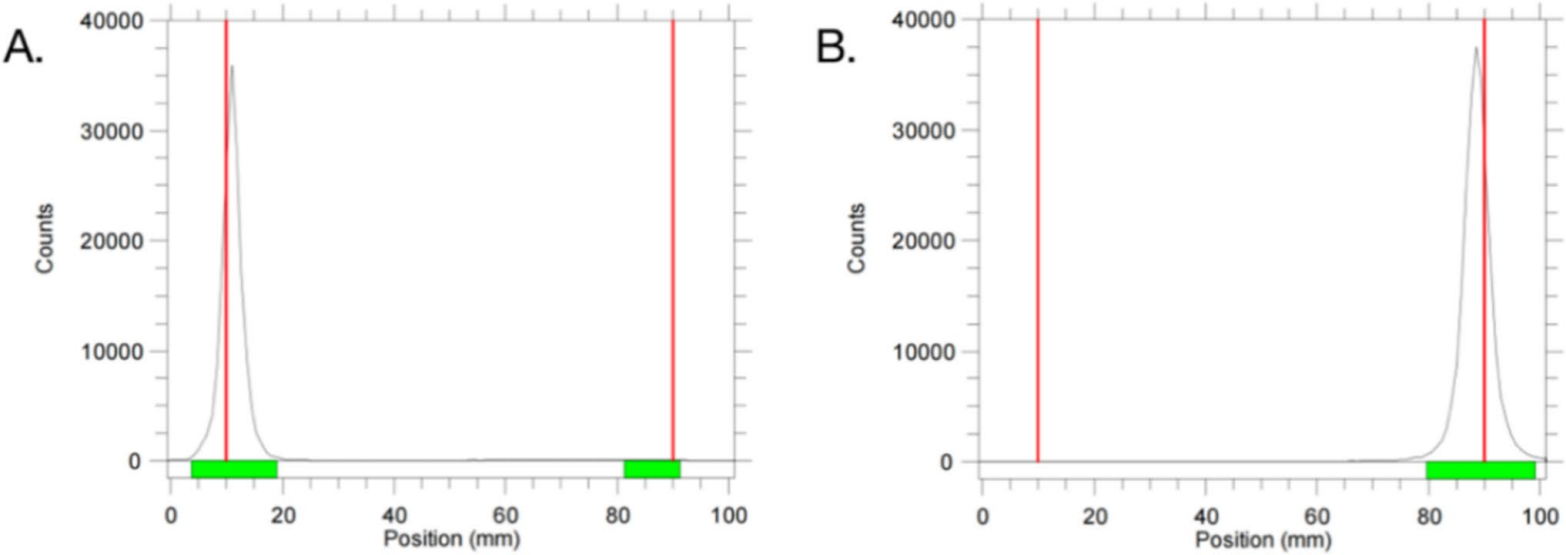
Examples of ITLC for ^99m^Tc-Z_IGF1R:4551_-GGGC and ^99m^TcO_4_^−^

The labeling efficiency of ^99m^Tc-Z_IGF1R:4551_-GGGC was determined using ITLC, with results depicted in Fig. 3. Figure 3A shows the emission peaks of ^99m^Tc-Z_IGF1R:4551_-GGGC and ^99m^TcO_4_^−^, respectively, from left to right, while Fig. 3B only shows the emission peaks of ^99m^TcO_4_^−^, and the labeling efficiency of ^99m^Tc-Z_IGF1R:4551_-GGGC is 99.48% (the green band corresponds to the location of the emission peaks). Repeated syntheses of ^99m^Tc-Z_IGF1R:4551_-GGGC consistently yielded labeling rates above 95.0%.

### Specific identification *in vivo*

**Fig. 4:**
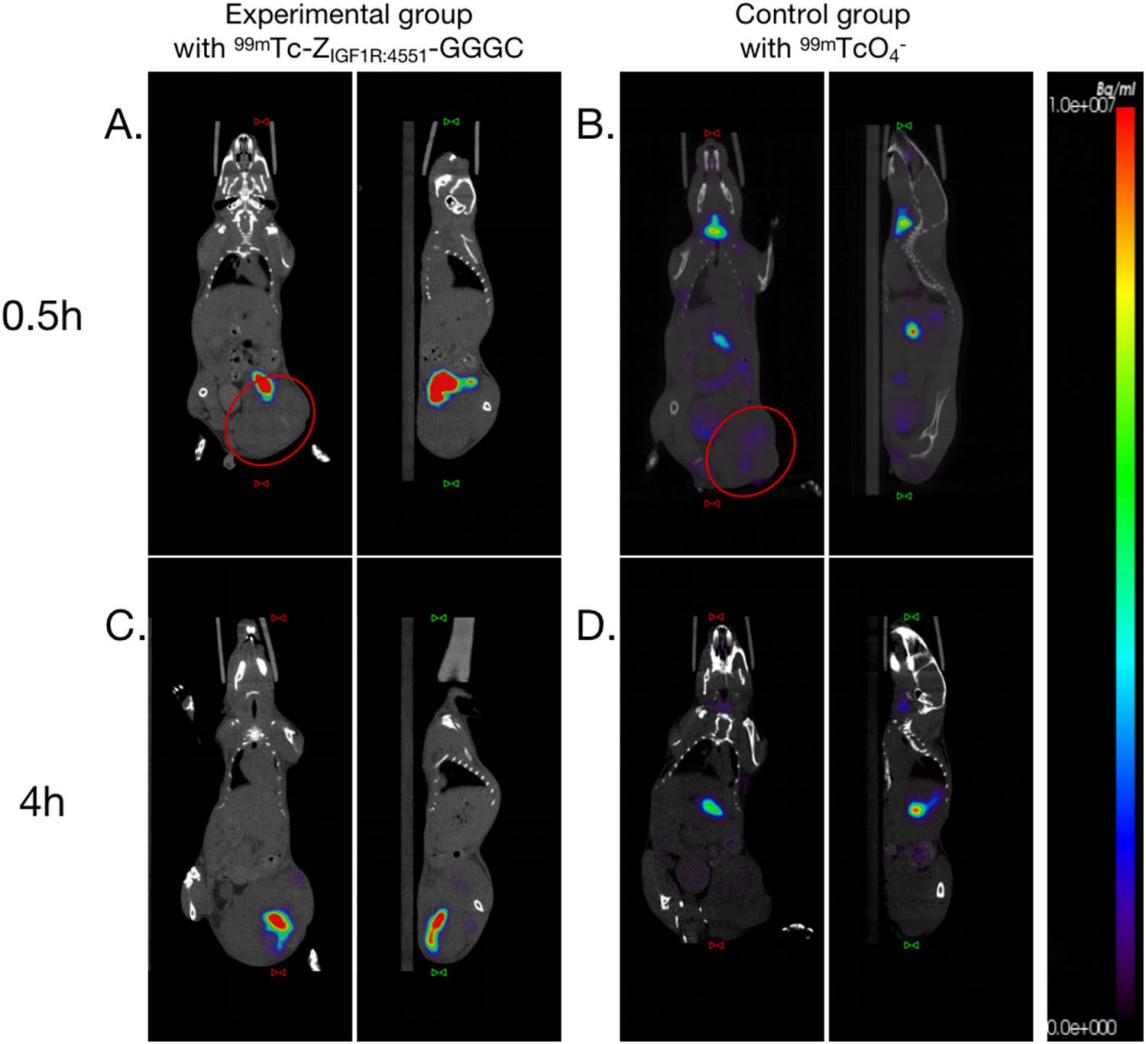
Imaging of tumor-bearing mice in the experimental group and control group. A and C SPECT/CT images of the experimental group 0.5 h and 4 h after injection of ^99m^Tc-Z_IGF1R:4551_-GGGC, respectively. B and D SPECT/CT images of the control group 0.5 h and 4 h after injection of ^99m^TcO_4_.^−^, respectively. (In the red box is the tumor)

Figure 4A shows the 0.5 h post-injection imaging of the B16F10 tumor-bearing mice in the experimental group. The tracer, ^99m^Tc-Z_IGF1R:4551_-GGGC, predominantly localized in and around the tumor (injection site). At 4 h post-injection, the tracer remained primarily within the tumor, centralizing in the tumor core as shown in Fig. 4C. There was no uptake of the tracer by the thyroid at either 0.5 h or 4 h. Control group imaging at 0.5 h (Fig. 4B) showed ^99m^TcO_4_^−^ distribution mainly in the thyroid, digestive tract, bladder, and tumor. After 4 h (Fig. 4D), the tracer predominantly localized in the digestive tract, with reduced concentrations in the thyroid, bladder, and tumor.

### Weight and serological

**Fig. 5:**
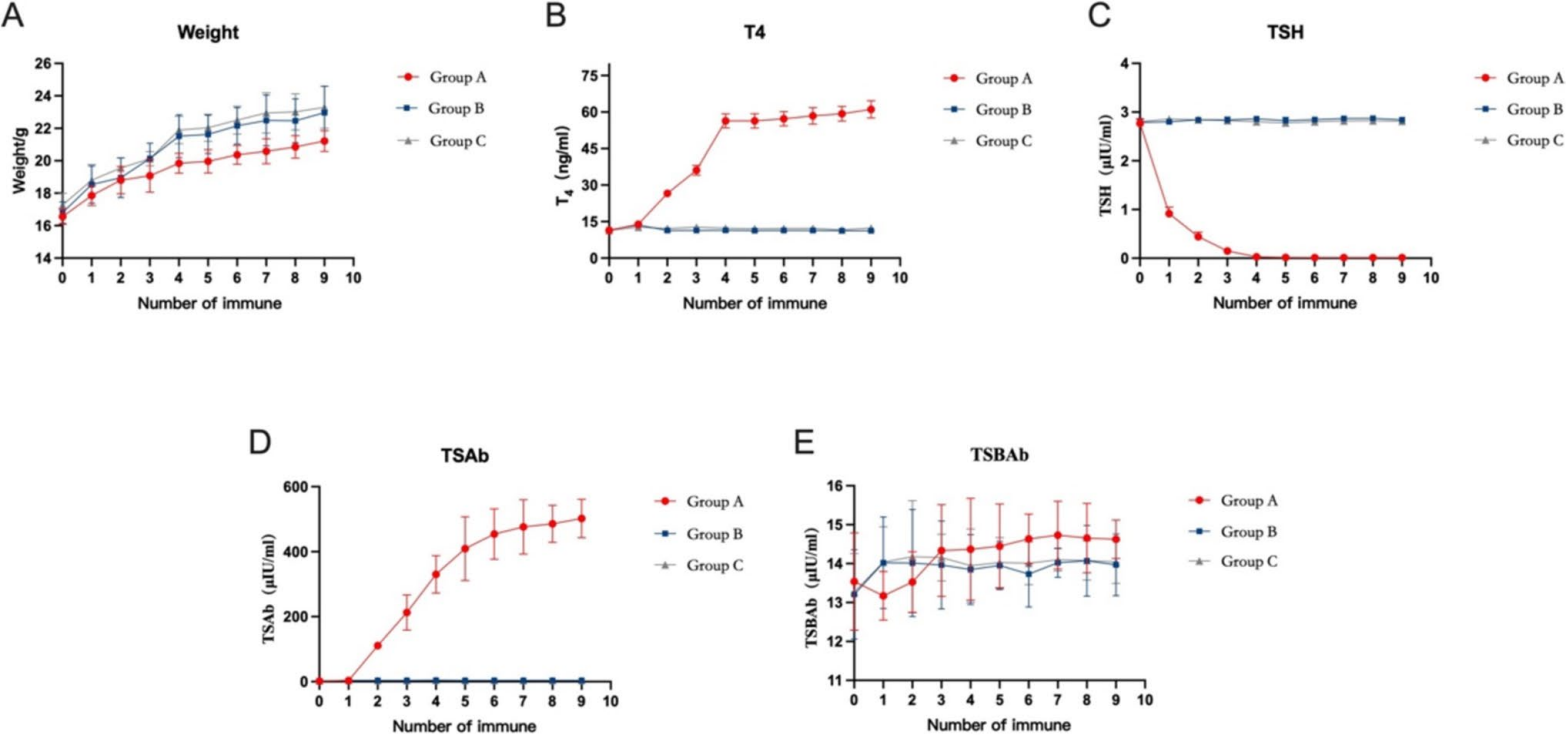
Weight and serological changes in each group

The baseline (before immunization) values of T4, TSH, TSAb and TSBAb were 11.37 ± 0.56 ng/mL, 2.77 ± 0.09 µIU /mL, 1.62 ± 0.25 µIU/mL, and 13.54 ± 1.25 µIU/mL in group A; 11.55 ± 0.64 ng/mL, 2.79 ± 0.08 µIU/mL, 1.78 ± 0.32 µIU/mL, and 13.21 ± 1.15 µIU/mL in group B; and 11.37 ± 0.94 µIU/mL, 2.81 ± 0.06 µIU/mL, 2.03 ± 0.32 µIU/mL, and 13.27 ± 0.99 µIU/mL in group C, respectively. These baseline values did not differ significantly (*P* > 0.05) among groups A, B, and C. After the second immunization, the T4 in group A was significantly higher than that in groups B and C (26.58 ± 1.06 ng/ml vs 11.35 ± 0.70 ng/ml, 12.17 ± 1.03 ng/ml) (*P* < 0.05, Fig. 5B). After the first immunization, the TSH in group A was significantly lower than that in groups B and C (0.91 ± 0.14 μIU/ml vs 2.80 ± 0.03 μIU/ml, 2.86 ± 0.03 μIU/ml) (*P* < 0.05, Fig. 5C). After the second immunization, the TSAb in group A was significantly higher than that in groups B and C (110.7 ± 9.51 μIU/ml vs 3.70 ± 0.49 μIU/ml, 3.82 ± 0.56 μIU/ml) (*P* < 0.05, Fig. 5D). During the immunization period, there was no significant difference in TSBAb among the groups A, B and C (*P* > 0.05, Fig. 5E); there were no significant differences in T4, TSH and TSAb between groups B and C (*P* > 0.05), T4 and TSAb in groups B and C were always lower than those in group A, and TSH was higher than those in group A (Fig. 5B, C, D).

### ^***99m***^***TcO***_***4***_^***−***^*** thyroid imaging and ocular MRI***

**Fig. 6:**
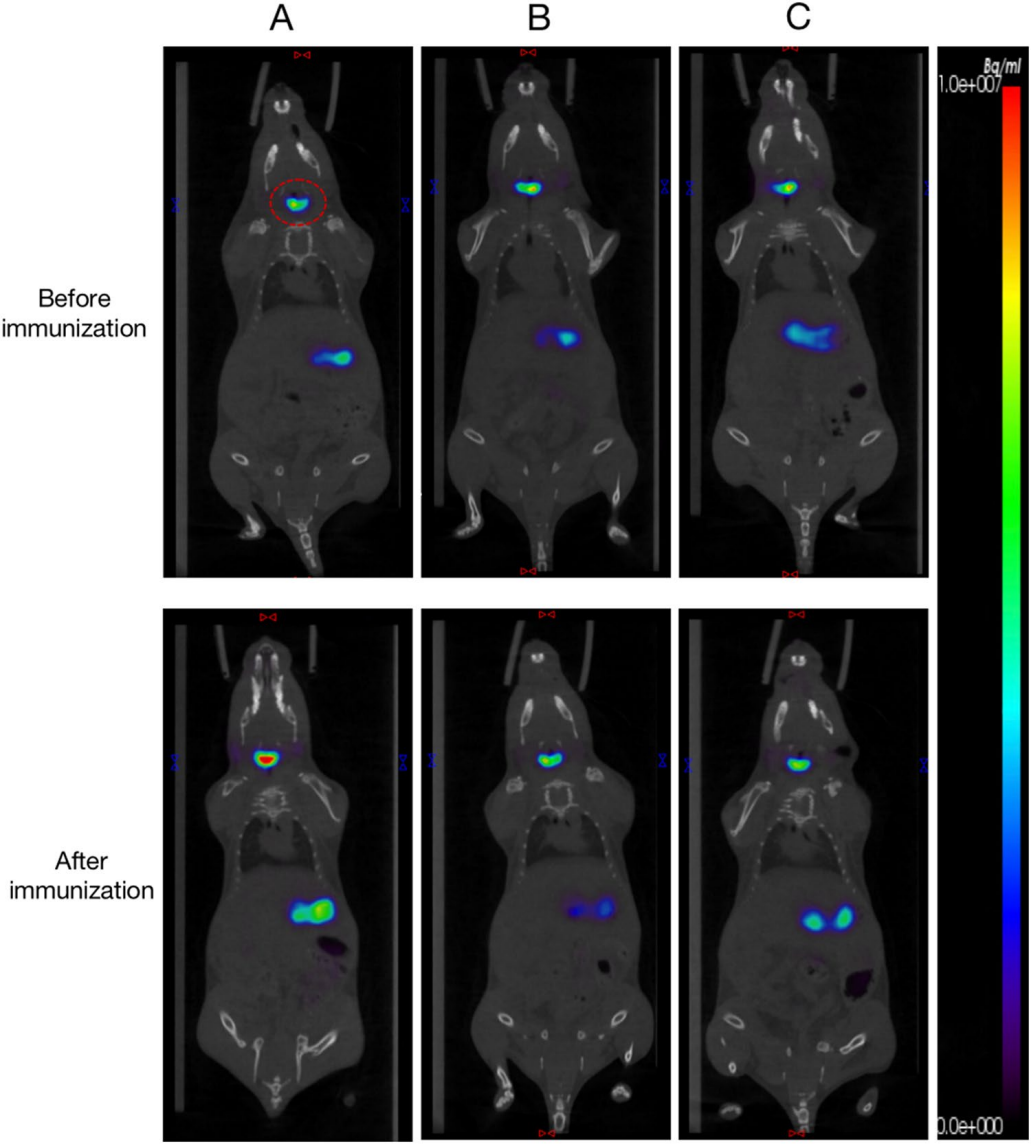
Thyroid SPECT/CT imaging. **A** Thyroid imaging before and after immunization in group A. **B** Thyroid imaging before and after immunization in group B. **C** Thyroid imaging at weeks 0 and 18 of group C (red box represents thyroid)

Prior to immunization, ^99m^TcO_4_^−^ thyroid imaging showed similar tracer uptake in all groups. Post-final immunization imaging revealed a significant increase in ^99m^TcO_4_^−^ uptake in group A compared to pre-immunization levels and to groups B and C. Groups B and C showed no significant change in tracer uptake, and no notable differences between them were observed (Fig. 6).

**Fig. 7:**
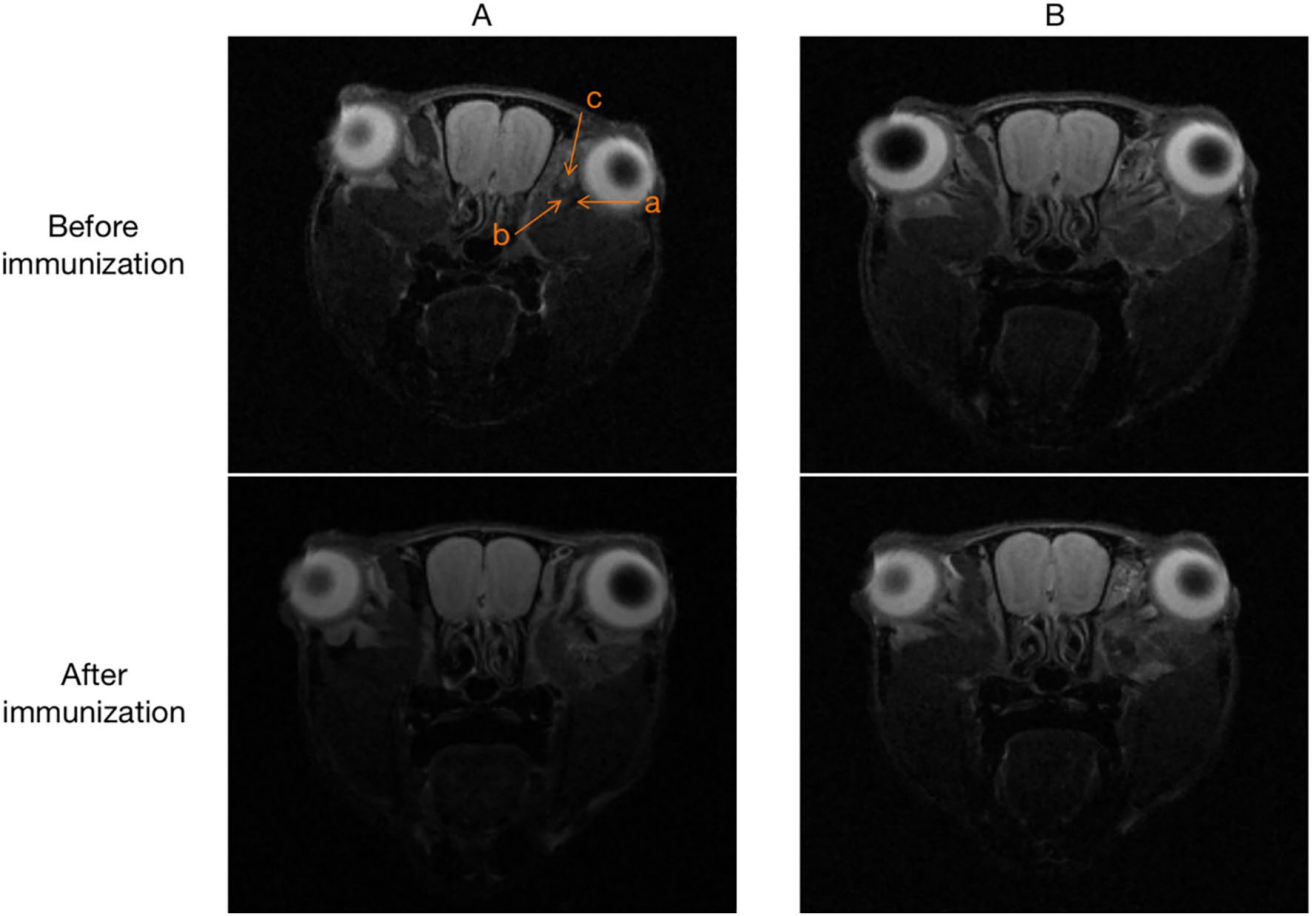
Positive sagittal position of ocular MRI T2. A Group A imaging before and after immunization: **a** eye muscle tissue with slightly low-signal, **b** optic nerve surrounded by cerebrospinal fluid with high signal, **c** harderian glands with high signal; B group B imaging before and after immunization

Orbital MRI imaging was performed in groups A and B before the first immunization and 1 week after the last immunization (i.e., week 0 and week 18). The positive sagittal images showed eye muscle with low signal, and no significant difference was observed between the two groups before immunization, that is, no significant difference was observed between the eye muscle before immunization. After the end of the last immunization, MRI T2-weighted phase was performed again, and it was found that the range of low signal area in group A was expanded compared with that before immunization and during the same period of group B, that is, the eye muscle in group A was thicker than that before immunization and during the same period of group B (Fig. 7).

### ***Specific imaging of ***^***99m***^***Tc-Z***_***IGF1R:4551***_***-GGGC***

**Fig. 8:**
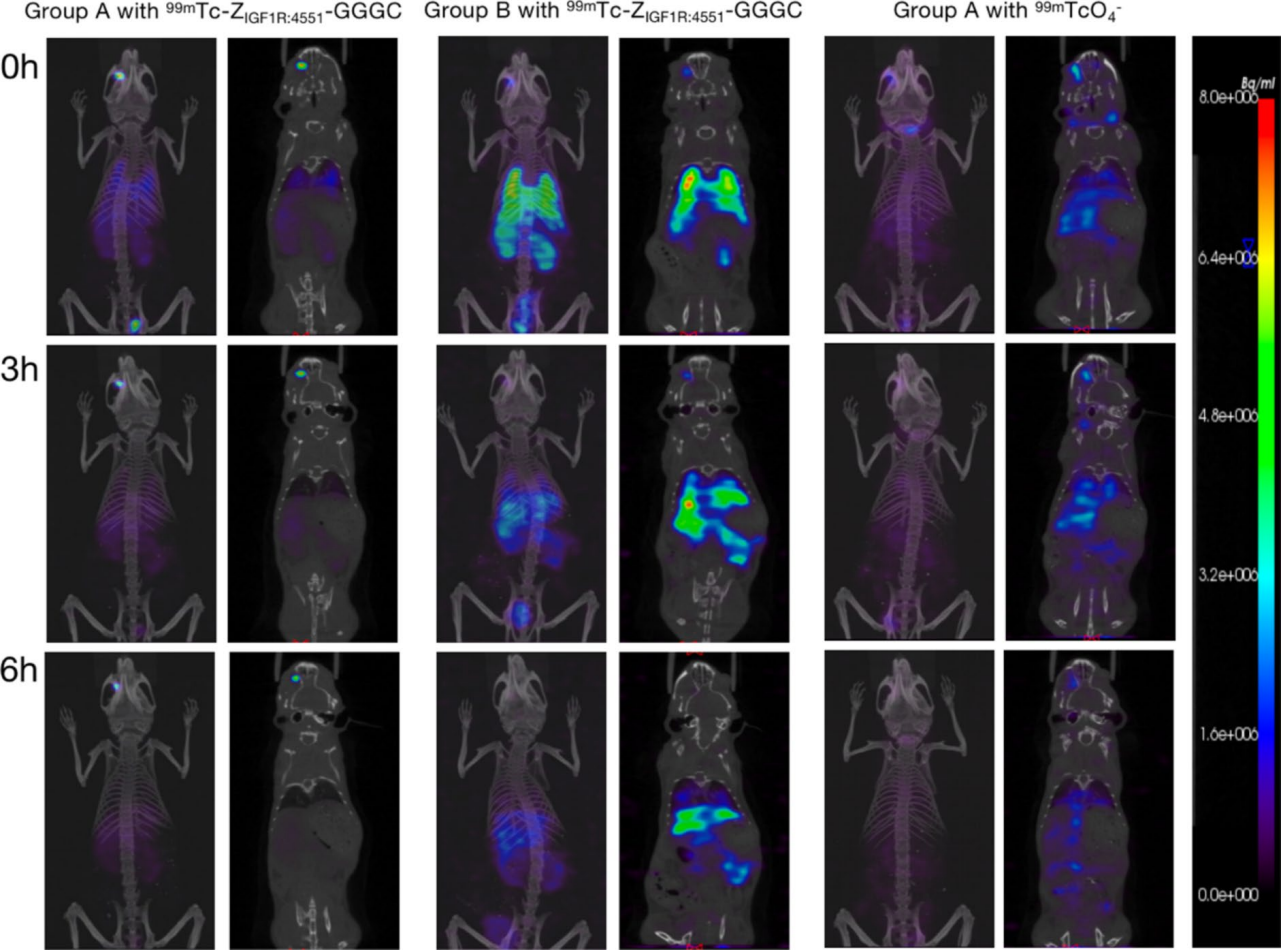
^99m^Tc-Z_IGF1R:4551_-GGGC imaging of groups A and B and ^99m^TcO_4_^−^ imaging of group A

SPECT imaging in a portion of group A was acquired at 0, 3, and 6 h after intravenous (right inner canthal vein) administration of ^99m^Tc-Z_IGF1R:4551_-GGGC (Fig. 8). Initial imaging revealed tracer visibility in the right retrobulbar tissue, lungs, liver, kidneys, digestive tract, and bladder, with a notable absence in the thyroid. The concentration in the right retrobulbar tissue was significantly higher than other areas. As time progressed, the tracer concentration in the retrobulbar tissue gradually decreased and narrowed at 3 and 6 h. At 6 h, the tracer was still visible in the retrobulbar tissue, while its presence in the lungs, liver, kidneys, digestive tract, and bladder significantly diminished. The thyroid was not imaged throughout. All the 8 mice in the experimental group had the above imaging characteristics.

Likewise, SPECT imaging in group B was acquired at 0, 3, and 6 h after intravenous administration of ^99m^Tc-Z_IGF1R:4551_-GGGC (Fig. 8). Initial imaging showed tracer accumulation in the right retrobulbar tissue, lungs, liver, and kidneys, digestive tract and bladder. However, the tracer concentration in the right retrobulbar tissue was markedly lower than in group A, with higher concentrations in other areas, particularly the lungs. After 3 and 6 h, the concentration and range in the retrobulbar tissue of group B decreased significantly, with no notable imaging observed after 6 h. The thyroid remained unimaged.

The imaging in another part of group A, imaging was acquired at 0.3.6 h after intravenous administration of ^99m^TcO_4_^−^ (Fig. 8). At 0 h, the tracer was visible in the right retrobulbar tissue, thyroid, digestive tract, and bladder, with a significantly stronger concentration in the thyroid. The concentration and scope in the retrobulbar tissue markedly decreased and narrowed over time. At 6 h, the retrobulbar tissue was no longer significantly imaged, while the tracer concentration in the thyroid gradually diminished yet remained visible.

The MIP fusion images and SPECT/CT fusion images obtained by groups A and B after injection of ^99m^Tc-Z_IGF1R:4551_-GGGC and group A after injection of ^99m^TcO4^−^ from left to right, respectively, from top to bottom represent different imaging times. In group A, the concentration degree of the tracer was significantly higher in the right retrobulbar tissue than in other parts, and the retrobulbar tissue was still visible at 6 h. The concentration of tracer in the right retrobulbar tissue of group B was significantly lower than that of group A. No thyroid imaging was observed in groups A and B after the injection of ^99m^Tc-Z_IGF1R:4551_-GGGC. After injection of ^99m^TcO4^−^ in group A, the imaging was most obvious in the thyroid, and no clear imaging was observed in the retrobulbar tissue at 6 h.

### Pathology of thyroid and retrobulbar tissue

**Fig. 9:**
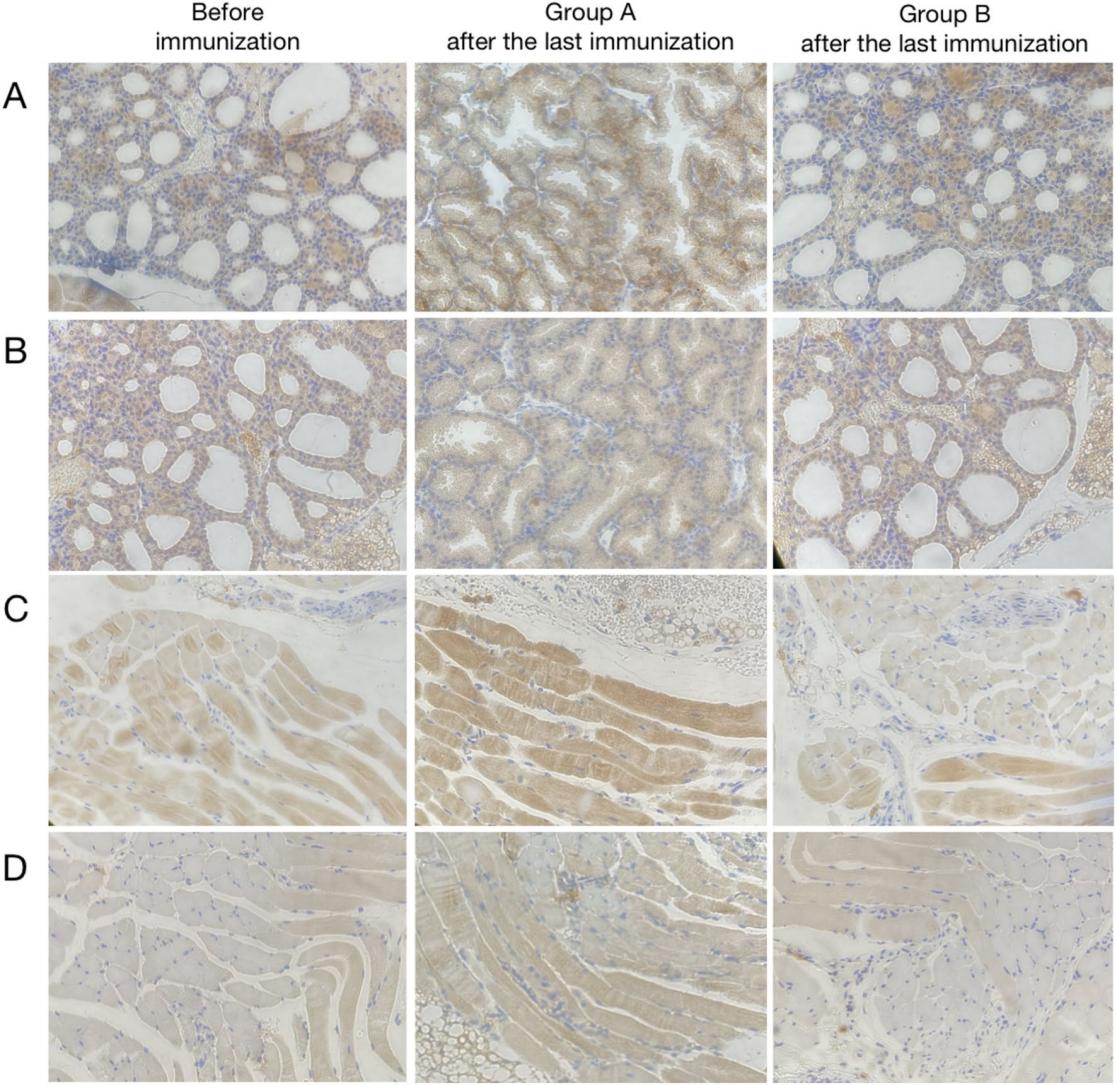
Immunohistochemical analysis of thyroid and retrobulbar tissue. **A** The expression of TSHR in thyroid tissues of each group; **B** the expression of IGF-1R in thyroid tissues of each group; **C** the expression of TSHR in retrobulbar tissues of each group; **D** the expression of IGF-1R in retrobulbar tissues of each group

TSHR and IGF-1R immunohistochemical analyses were performed on thyroid and retrobulbar tissues of groups A and B. After the last immunization, compared with before immunization and group B, TSHR and IGF-1R were significantly expressed in the thyroid tissue of group A (Fig. 9). Similarly, TSHR and IGF-1R in the retrobulbous tissues of group A were also significantly expressed (Fig. 9).

## Discussion

At present, the clinical evaluation of GO and the treatment choice is mainly based on the disease activity and severity [12]. Common assessment methods include Clinical activity score (CAS) and imaging. Most of the examination methods are mainly applied to the assessment of moderate-severe or active stage of GO, but cannot be used to evaluate the early lesions of GO without morphological changes. ^99m^Tc-DTPA orbital SPECT/CT imaging can directly reflect the inflammatory response during the GO active stage [13] and can evaluate the inflammatory response or the efficacy of the imaging agent uptake [14, 15]. ^18^F-FDG PET/CT can also detect the inflammation sites. Kuo et al. applied it for the first time to detect the inflammation of GO, and the results showed that the extraocular muscle uptake of tracer increased in GO patients, but this test is not routinely recommended for clinical examination [16–19]. In addition, octreotide is a somatostatin analog that binds specifically to the somatostatin receptor (SSTR). Studies have found that SSTR is expressed in T lymphocytes of ocular and active fibroblasts in GO patients, and Postema et al. applied octreotide imaging for the first time in GO [20]. However, there is a lack of imaging methods for the early and inactive stages of GO, so it is important to explore imaging methods for the early diagnosis and treatment of it.

Previous studies have shown that TSHR plays an important role in the pathogenesis of GD and IGR-1R is the initiator and pathogenic factor in GO [2, 3]. In addition, IGF-1R is highly over-expressed on orbital fibroblasts; therefore, in the present study, we explored the potential of IGR-1R-based imaging in GO which has not been investigated previously. IGF-1R is involved in the pathogenesis of various tumors, and its high expression is associated with metastasis and treatment resistance [7]. Therefore, IGF-1R is often used as a therapeutic target for prostate cancer, breast cancer, and melanoma [21]. Tolmachev et al. constructed a probe with affinity to IGF-1R and successfully conducted targeted imaging [6].

For animal imaging, we explored the construction of GO models. The first GO model was constructed in 2013 by Moshkelgosha’s team, which used the TSHRα for immunization [8]. With the progress of research, it is found that TSHR and IGF-1R have a co-localization phenomenon and synergistic function, which is an important mechanism of GO [1–4, 9]. It was confirmed by Krieger et al. that the simultaneous action of TSH and IGF-1 produced more hyaluronic acid than that of TSH or IGF-1 alone [22]. Based on the previous results, we further explore the construction method of the GO model. First, the IGF-1Rα subunit recombinant plasmid pcDNA3.1/IGF-1Rα was successfully extracted [10]. Then, based on the mechanism of TSHR and IGF-1R, we injected the above two recombinant plasmids into BALB/c mice at the same time, and successfully constructed GO models [11].

In the present study, we used this noble SPECT tracer (^99m^Tc-Z_IGF1R:4551_-GGGC) in imaging GO animal tumor models to evaluate its sensitivity and specificity for targeting IGF-1R. The results showed that the uptake of ^99m^Tc-Z_IGF1R:4551_-GGGC in GO-induced animal models was significantly higher than in the normal mice. In addition, the tracer also showed longer retention in the area localizing GO tissue. The uptake of ^99m^Tc-Z_IGF1R:4551_-GGGC was still present in the retrobulbous site 6 h after injection, while no significant uptake was seen in the normal mice 3 h after injection, and basically no uptake at 6 h. In addition, GO mice were injected with ^99m^TcO_4_^−^ under the same conditions, and the results showed that the uptake of ^99m^TcO_4_^−^ in GO mice was mainly concentrated in the thyroid, and partly concentrated in the digestive tract and bladder and other organs. The above results indicate that ^99m^Tc-Z_IGF1R:4551_-GGGC has affinity, specificity and serum stability, and can firmly bind to ^99m^Tc, so it can perform sensitive and specific imaging on GO mice.

However, there are still some shortcomings in this study. First, for animal models, no systematic health assessment was performed on mice after shortening the immune interval, such as blood routine, blood glucose, and pathological analysis of related organs such as the heart and liver. In addition, the serum indicators associated with GD were mainly tested, and serological tests for IGF-1R were not performed. Second, due to the chemical nature of peptides, if injected into the body through the tail vein, the peptides will first enter the liver through the blood circulation, which will affect the exploration of targeting. Therefore, the tail vein injection is not adopted. Finally, although IGF-1R-specific imaging was successfully performed on active GO mice in this study, no imaging was performed at earlier time points before the onset of symptoms of active GO. In light of this limitation, our laboratory plans to address this in subsequent research.

In conclusion, the GO model was developed successfully which could be used conveniently for validating the affinity of ^99m^Tc-Z_IGF1R:4551_-GGGC as a noble SPECT tracer for targeting IGF-1R over-expressed in GO and GD patho-pathologies. This SPECT imaging tracer holds great potential for the detection of GO and GD at an early stage and also for response evaluation to IGF-1R-targeted therapies. However, the tracer needs a detailed evaluation by further extensive pre-clinical studies.
